# Supplementary figures and images for: Witnessing the structural evolution of an RNA enzyme
Source: eLife. 2021 Sep 9;10:e71557. doi: 10.7554/eLife.71557 (PMC8460264; doi:10.7554/eLife.71557)

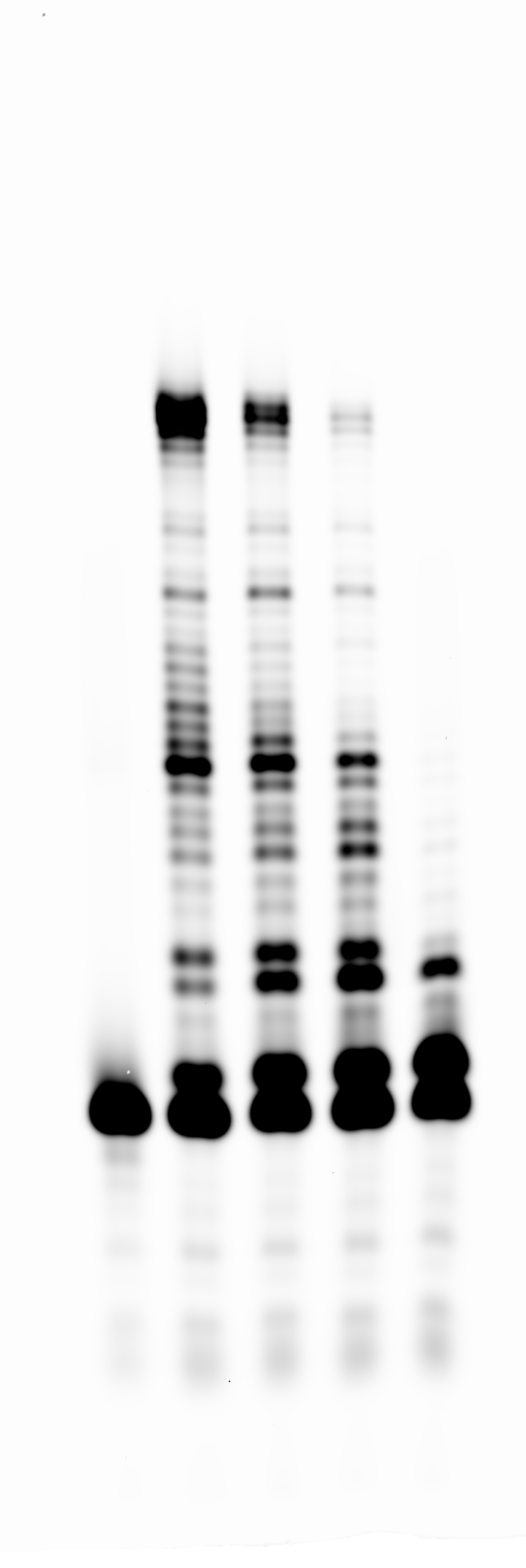

Supplement: Figure 1—source data 1. [file elife-71557-fig1-data1.zip › Figure 1 source data 1a.tif]

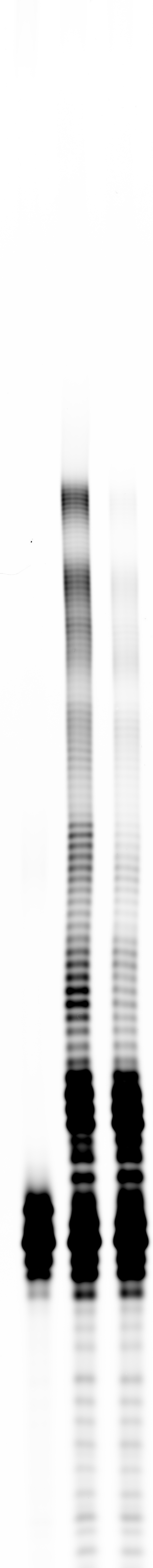

Supplement: Figure 1—source data 1. [file elife-71557-fig1-data1.zip › Figure 1 source data 1c.tif]

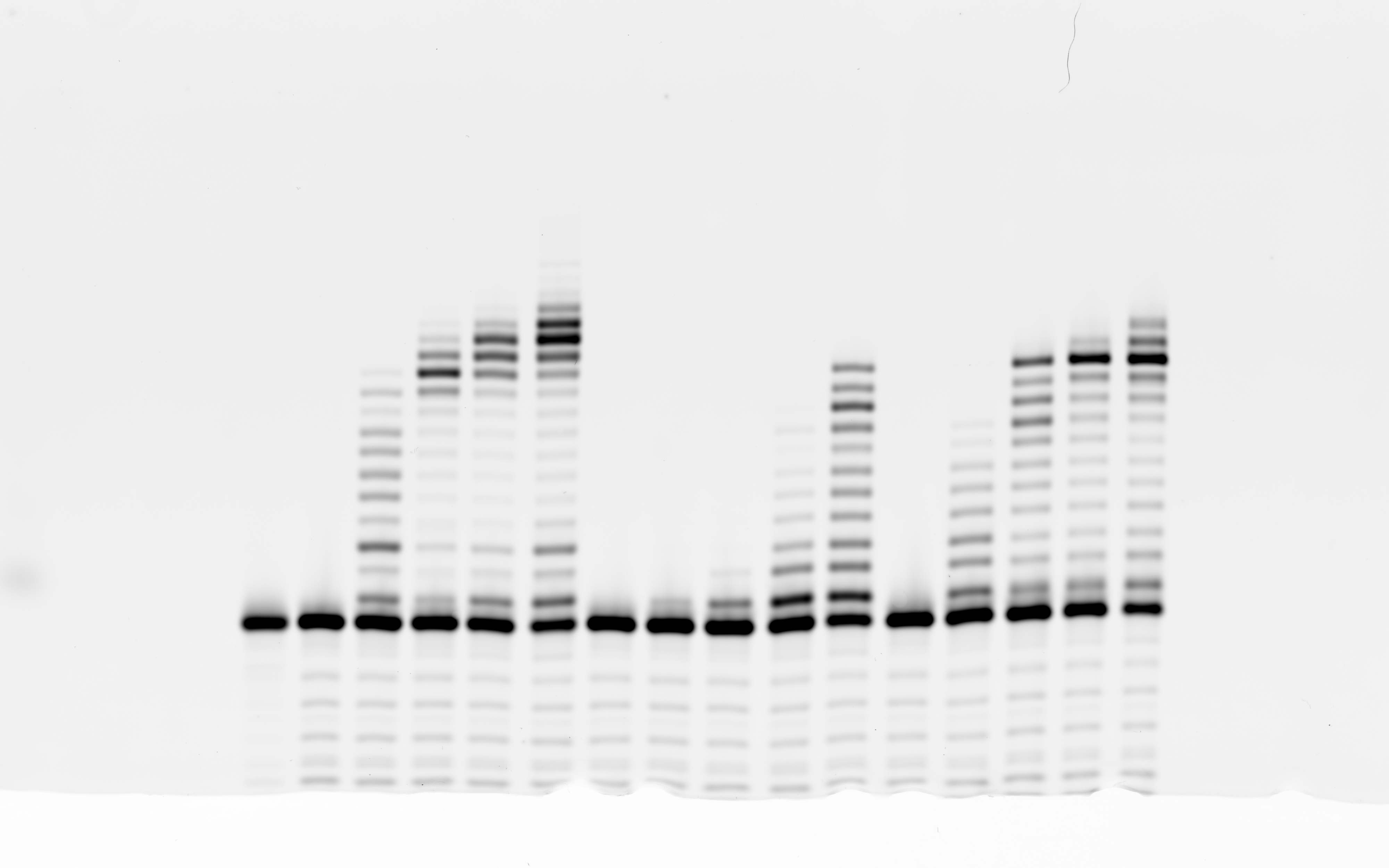

Supplement: Figure 2—figure supplement 2—source data 1. [file elife-71557-fig2-figsupp2-data1.zip › Figure 2 - figure supplement 2 - source data 1a.tif]

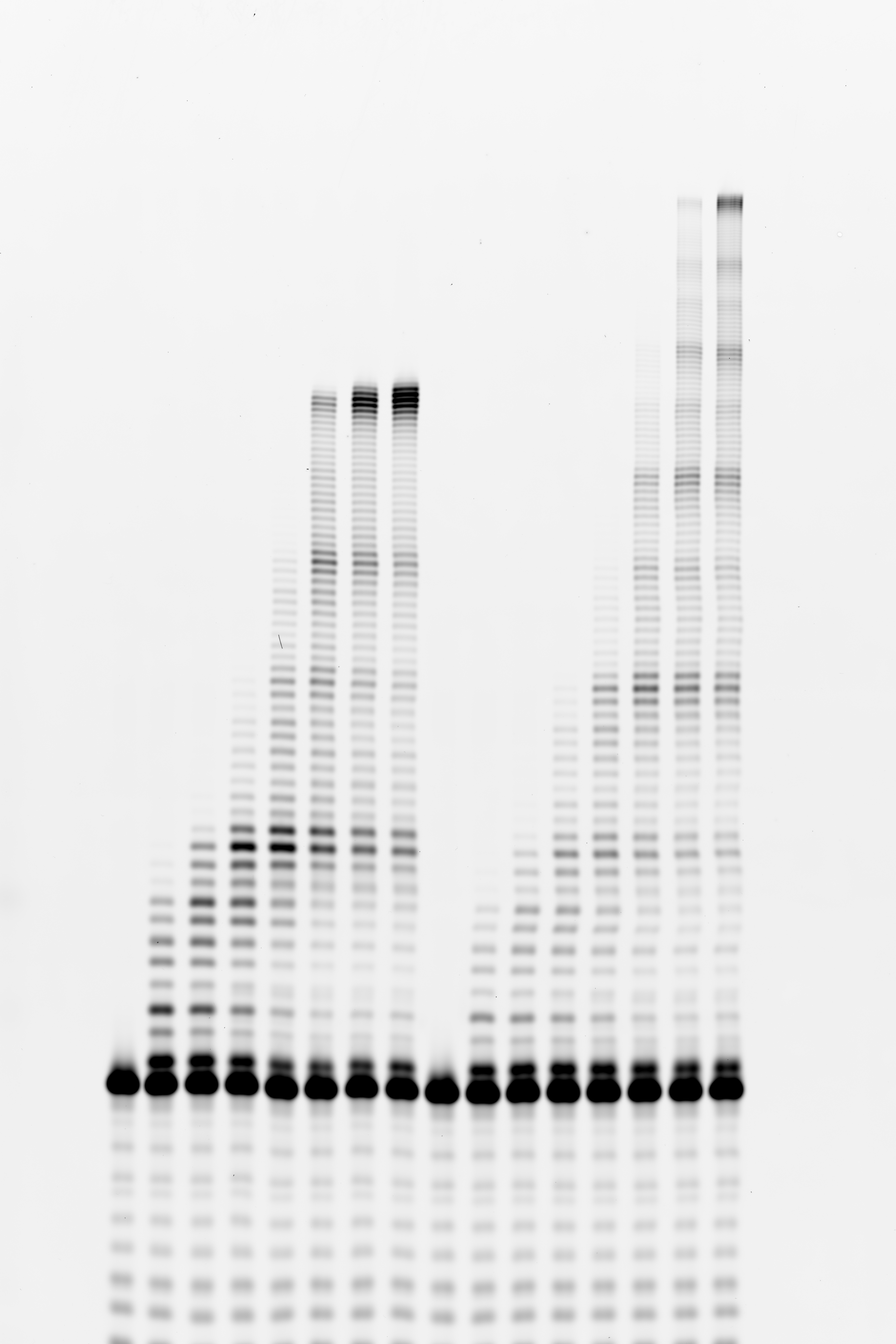

Supplement: Figure 3—figure supplement 2—source data 1. [file elife-71557-fig3-figsupp2-data1.zip › Figure 3 - figure supplement 2 - source data 1a.tif]

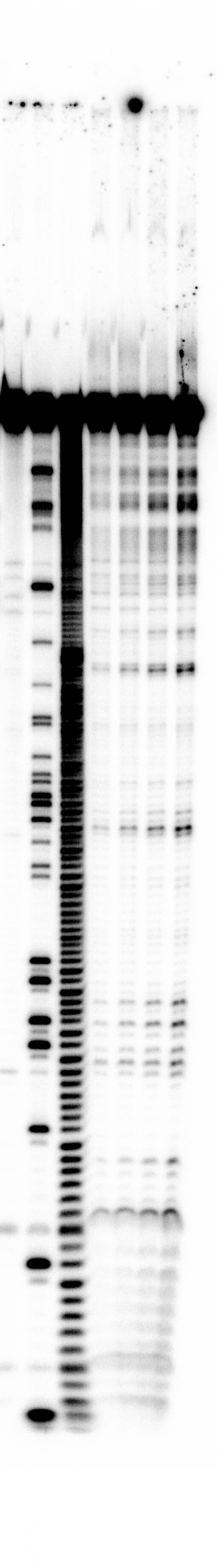

Supplement: Figure 4—source data 1. [file elife-71557-fig4-data1.zip › Figure 4 - source data 1a.tif]

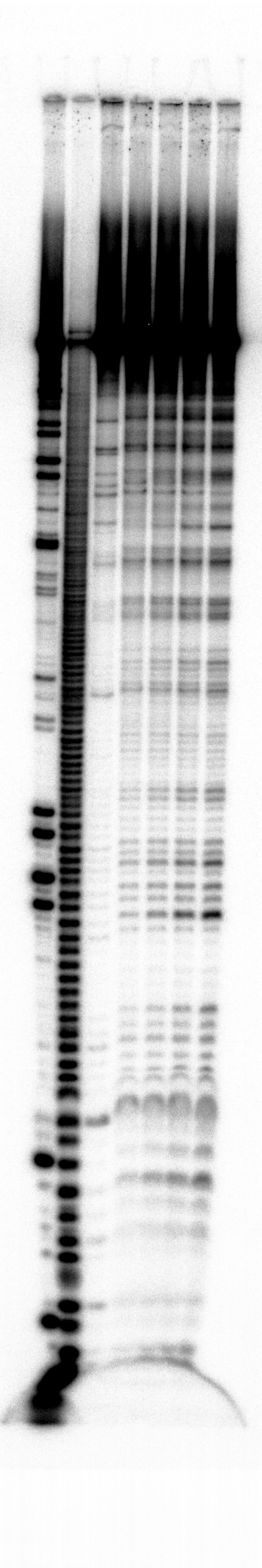

Supplement: Figure 4—source data 1. [file elife-71557-fig4-data1.zip › Figure 4 - source data 1b.tif]

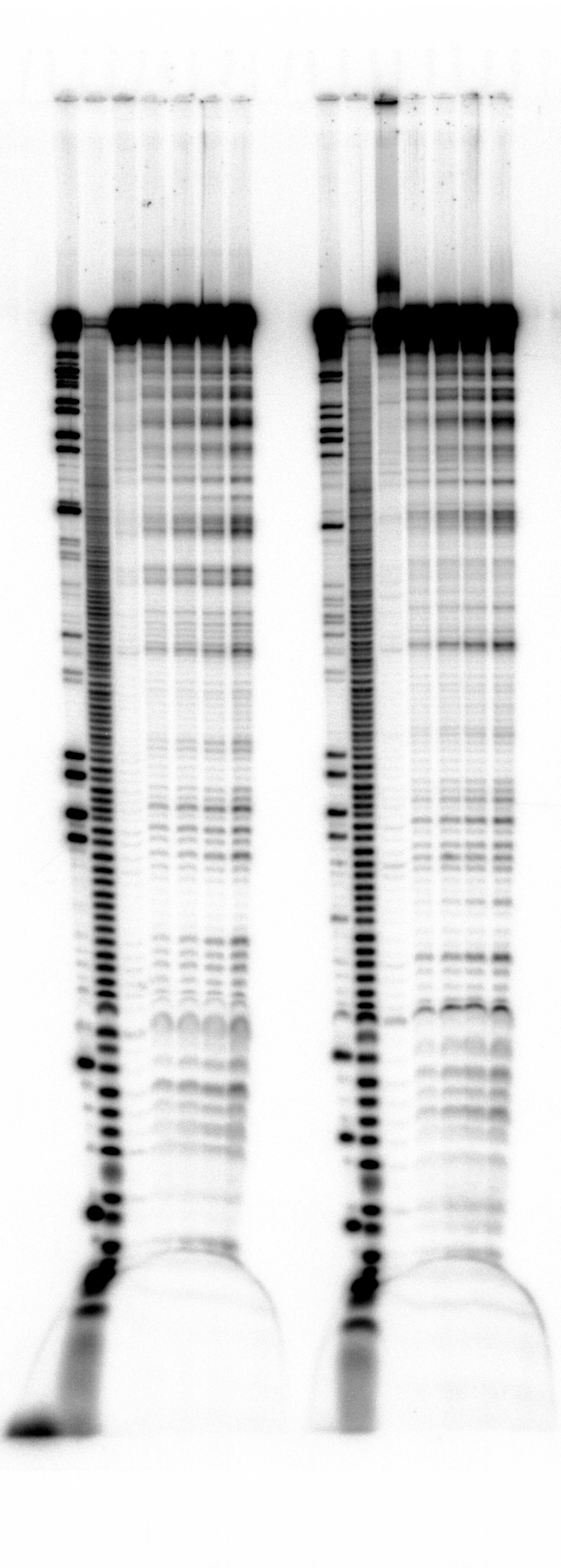

Supplement: Figure 4—figure supplement 1—source data 1. [file elife-71557-fig4-figsupp1-data1.zip › Figure 4 - figure supplement 1 - source data 1a.tif]
