# Supplementary material for: Witnessing the structural evolution of an RNA enzyme
Source: eLife. 2021 Sep 9;10:e71557. doi: 10.7554/eLife.71557 (PMC8460264; doi:10.7554/eLife.71557)
Supplement: Supplementary file 1. — The third column indicates the number of nucleoside 5’-triphosphates (NTPs) to be added in order to meet the selection criterion. ‘+’ indicates PCR mutagenesis; ‘10%’ indicates random mutagenesis of the 38-6 polymerase at 10% degeneracy per position. [file elife-71557-supp1.docx]

**Supplementary file 1. Parameters for directed evolution of polymerase ribozymes.**

| Round | Selection method | +NTPs | Time (h) | [Mg^2+^] (mM) | Mutagenesis |
| --- | --- | --- | --- | --- | --- |
| 1 | gel-shift | 20 | 24 | 200 |  |
| 2 | " | " | " | " |  |
| 3 | " | " | " | " |  |
| 4 | " | " | " | " | + |
| 5 | vitamin B_12_ aptamer | 12 | 4 | " |  |
| 6 | " | " | " | " |  |
| 7 | " | " | " | " |  |
| 8 | gel-shift | 18 | 24 | " |  |
| 9 | " | " | " | " |  |
| 10 | " | " | " | " |  |
| 11 | " | " | " | " |  |
| 12 | " | " | " | " | + |
| 13 | " | 30 | 2 | " |  |
| 14 | " | " | " | " |  |
| 15 | " | " | " | " |  |
| 16 | " | " | " | " |  |
| 17 | gel-shift & GTP aptamer | " | " | " | + |
| 18 | gel-shift | 32 | 6 | " |  |
| 19 | " | " | 1 | " |  |
| 20 | " | " | 0.25 | " |  |
| 21 | " | 40 | " | " |  |
| 22 | gel-shift & GTP aptamer | 30 | " | " | + |
| 23 | " | " | " | " |  |
| 24 | " | " | " | " |  |
| 25 | hammerhead | 33 | 2 | " |  |
| 26 | " | " | 0.33 | " |  |
| 27 | " | " | " | " |  |
| 28 | " | " | " | " |  |
| 29 | " | " | " | " |  |
| 30 | " | " | " | " |  |
| 31 | gel-shift & hammerhead | " | " | " |  |
| 32 | " | " | " | " |  |
| 33 | " | " | " | " |  |
| 34 | " | " | 0.08 | " |  |
| 35 | " | " | " | " |  |
| 36 | " | " | " | " |  |
| 37 | " | " | " | " |  |
| 38 | " | " | " | " | 10% |
| 39 | urea wash | 28 | 15 | " |  |
| 40 | " | " | 2.5 | " |  |
| 41 | gel-shift & hammerhead | 33 | 20 | " |  |
| 42 | " | " | 2 | " |  |
| 43 | " | " | " | " | + |
| 44 | " | " | 1 | " | + |
| 45 | " | " | 0.4 | 50 | + |
| 46 | " | " | 4 | " | + |
| 47 | " | " | 1 | " | + |
| 48 | " | " | 0.25 | " | + |
| 49 | " | " | " | " | + |
| 50 | " | " | " | " | + |
| 51 | " | " | 1 | 25 |  |
| 52 | " | " | 24 | 50 |  |

The third column indicates the number of NTPs to be added in order to meet the selection criterion. “+” indicates PCR mutagenesis; “10%” indicates random mutagenesis of the 38-6 polymerase at 10% degeneracy per position.
