# Supplementary material for: Witnessing the structural evolution of an RNA enzyme
Source: eLife. 2021 Sep 9;10:e71557. doi: 10.7554/eLife.71557 (PMC8460264; doi:10.7554/eLife.71557)
Supplement: Supplementary file 2. — The 52-2 polymerase was used to synthesize the hammerhead and class I ligase ribozymes (see Supplementary file 4 for sequences). The hammerhead was synthesized in the presence of either 200 or 50 mM Mg2+ and the ligase was synthesized in the presence of 200 mM Mg2+. The full-length products were analyzed by deep sequencing to obtain the frequencies of each type of mutation. The average fidelity was calculated as the geometric mean of the fidelities for each templating nucleobase, which gave values of 91.7% or 94.4% for the hammerhead with either 200 or 50 mM Mg2+, respectively, and 84.1% for the ligase. Deletions were included as mutations and insertions were treated as a single mutation at the immediately upstream position. [file elife-71557-supp2.docx]

**Supplementary file 2. Fidelity of the 52-2 polymerase.**

| Product [Mg^2+^] | Expected | Observed | | | | | |
| --- | --- | --- | --- | --- | --- | --- | --- |
|  |  | A | C | G | U | Del | Ins |
| Hammerhead 200 mM | A | **85.9** | 1.6 | 8.2 | 0.4 | 3.8 | 0.3 |
|  | C | 0.3 | **90.1** | 0.7 | 8.4 | 0.6 | 0.2 |
|  | G | 0.4 | 0.5 | **98.3** | 0.1 | 0.7 | 0.2 |
|  | U | 0.6 | 3.9 | 1.3 | **92.9** | 1.4 | 0.2 |
| Hammerhead 50 mM | A | **90.4** | 0.8 | 5.4 | 0.2 | 3.2 | 0.1 |
|  | C | 0.1 | **93.4** | 0.2 | 6.0 | 0.3 | 0.0 |
|  | G | 0.1 | 0.2 | **99.2** | 0.1 | 0.4 | 0.1 |
|  | U | 0.3 | 2.5 | 0.9 | **94.9** | 1.4 | 0.1 |
| Ligase 200 mM | A | **79.3** | 3.7 | 9.9 | 1.1 | 5.9 | 0.4 |
|  | C | 0.5 | **89.8** | 1.1 | 6.0 | 2.6 | 0.5 |
|  | G | 0.8 | 0.4 | **97.2** | 0.3 | 1.2 | 0.4 |
|  | U | 2.8 | 6.8 | 5.7 | **72.4** | 12.3 | 0.4 |

The 52-2 polymerase was used to synthesize the hammerhead and class I ligase ribozymes (see supplementary file 4 for sequences). The hammerhead was synthesized in the presence of either 200 or 50 mM Mg^2+^ and the ligase was synthesized in the presence of 200 mM Mg^2+^. The full-length products were analyzed by deep sequencing to obtain the frequencies of each type of mutation. The average fidelity was calculated as the geometric mean of the fidelities for each templating nucleobase, which gave values of 91.7% or 94.4% for the hammerhead with either 200 or 50 mM Mg^2+^, respectively, and 84.1% for the ligase. Deletions were included as mutations and insertions were treated as a single mutation at the immediately upstream position.
