# Supplementary material for: Witnessing the structural evolution of an RNA enzyme
Source: eLife. 2021 Sep 9;10:e71557. doi: 10.7554/eLife.71557 (PMC8460264; doi:10.7554/eLife.71557)
Supplement: Supplementary file 3. — Clusters representing >1% of the population in a given round are shown. Clusters that differ by only a single nucleotide outside the P8 stem are given the same name. Clusters that include the wild type, 24-3, 38-6, or 52-2 polymerase are given the name of that polymerase. [file elife-71557-supp3.docx]

**Supplementary file 3. Prevalent sequence clusters over the course of evolution.**

| Round | Cluster | Nucleotides 9–17 | Nucleotides 83–95 | Frequency (%) |
| --- | --- | --- | --- | --- |
| 6 | wild type | CCGCACAAA | AUAGCG--CCAAC | 91.2 |
| 6 | 6.1 | CCGCACAAA | AUGGCG--CCAAC | 1.2 |
| 8 | wild type | CCGCACAAA | AUAGCG--CCAAC | 64.0 |
| 8 | 8.1 | CCGCACAAA | CAAGCG--CCAAC | 4.8 |
| 8 | 6.1 | CCGCACAAA | AUGGCG--CCAAC | 3.5 |
| 8 | wild type | CCGCACAAA | AUAGCG--CCAUC | 2.1 |
| 8 | 8.2 | CCGCACAGA | AUGGCG--CCAAC | 1.6 |
| 8 | wild type | CCGCACAAA | ACAGCG--CCAGC | 1.6 |
| 8 | 8.3 | CCGCACAGA | AUAGCG--CCAAC | 1.5 |
| 8 | 8.4 | CCGCACAAA | AUAGCG--CCAGC | 1.5 |
| 8 | 8.5 | CCGCACGAA | AUAGCG--CCAAC | 1.1 |
| 8 | wild type | CCGCACAAA | AUAGCG--UCAAC | 1.1 |
| 11 | 11.1 | CCGCACUAA | AUAGUG--CCAAC | 27.1 |
| 11 | wild type | CCGCACAAA | AUAGCG--CCAAC | 24.8 |
| 11 | 11.2 | CCGCACAAA | AUGGCU--CGAAG | 16.1 |
| 11 | 24-3 | CCGCACGAA | AUCGUG--CCACC | 7.4 |
| 11 | 11.3 | CCGCACAAA | AUUGUG--CCACC | 6.1 |
| 11 | 8.1 | CCGCACAAA | CAAGCG--CCAAC | 2.4 |
| 11 | 11.1 | CCGCACUAA | AUAGUG--CCACC | 1.5 |
| 11 | 24-3 | CCGCACGAA | AUCGUG--CCAAC | 1.3 |
| 11 | 11.4 | CCGCACAAA | AUCGCG--CCACC | 1.2 |
| 11 | 11.5 | CCGCACAGA | ACUGUG--CCACC | 1.1 |
| 11 | 11.4 | CCGCACAAA | AUCGCG--CCAAC | 1.0 |
| 12 | 11.1 | CCGCACUAA | AUAGUG--CCAAC | 45.2 |
| 12 | 11.3 | CCGCACAAA | AUUGUG--CCACC | 13.7 |
| 12 | 24-3 | CCGCACGAA | AUCGUG--CCACC | 13.6 |
| 12 | wild type | CCGCACAAA | AUAGCG--CCAAC | 7.2 |
| 12 | 11.2 | CCGCACAAA | AUGGCU--CGAAG | 7.1 |
| 12 | wild type | CCGCACAAA | AUAGCG--CCAUC | 2.1 |
| 12 | 11.1 | CCGCACUAA | AUAGUG--CCACC | 1.6 |
| 12 | 24-3 | CCGCACGAA | AUCGUG--CCAAC | 1.1 |
| 14 | 24-3 | CCGCACGAA | AUCGUG--CCACC | 63.4 |
| 14 | 11.3 | CCGCACAAA | AUUGUG--CCACC | 10.1 |
| 14 | wild type | CCGCACAAA | AUAGCG--CCAAC | 9.3 |
| 14 | 11.2 | CCGCACAAA | AUGGCU--CGAAG | 6.6 |
| 14 | 11.1 | CCGCACUAA | AUAGUG--CCAAC | 2.5 |
| 14 | 24-3 | CCGCACGAA | AUCGUG--CCAAC | 2.1 |
| 14 | 14.1 | CCGCACAAA | -U-GUG--CCACC | 1.3 |
| 16 | 24-3 | CCGCACGAA | AUCGUG--CCACC | 73.1 |
| 16 | 14.1 | CCGCACAAA | -U-GUG--CCACC | 14.9 |
| 16 | 11.5 | CCGCACAGA | ACUGUG--CCACC | 1.7 |
| 16 | 24-3 | CCGCACGAA | AUCGUG--CCAUC | 1.4 |
| 18 | 24-3 | CCGCACGAA | AUCGUG--CCACC | 69.9 |
| 18 | 14.1 | CCGCACAAA | -U-GUG--CCACC | 16.2 |
| 18 | 24-3 | CCGCACGAA | AUCGUG--CCAUC | 2.2 |
| 18 | 11.3 | CCGCACAAA | AUUGUG--CCAAC | 2.1 |
| 18 | 18.1 | CCGCACGAA | UUCGUG--CCACC | 2.1 |
| 18 | 18.2 | CCGCACUAA | UUAGUG--CCACC | 1.0 |
| 18 | 18.1 | CCGCACGAA | UUCGUG--CCAUC | 1.0 |
| 21 | 24-3 | CCGCACGAA | AUCGUG--CCACC | 75.4 |
| 21 | 14.1 | CCGCACAAA | -U-GUG--CCACC | 18.3 |
| 21 | 14.1 | CCGCACAAA | AU-GUG--CCACC | 1.4 |
| 21 | 18.1 | CCGCACGAA | UUCGUG--CCACC | 1.1 |
| 24 | 24-3 | CCGCACGAA | AUCGUG--CCACC | 60.2 |
| 24 | 14.1 | CCGCACAAA | AU-GUG--CCACC | 31.3 |
| 24 | 24.1 | CCGCACGAA | AU-GUG--CCACC | 2.1 |
| 24 | 24.2 | CCGCACAAA | AUCGUG--CCACC | 2.0 |
| 24 | 14.1 | CCGCACAAA | ---GUG--CCACC | 1.1 |
| 27 | 24-3 | CCGCACGAA | AUCGUG--CCACC | 55.2 |
| 27 | 14.1 | CCGCACAAA | -U-GUG--CCACC | 31.1 |
| 27 | 24.1 | CCGCACGAA | -U-GUG--CCACC | 5.7 |
| 27 | 18.2 | CCGCACUAA | UUAGUG--CCACC | 2.1 |
| 27 | 24.2 | CCGCACAAA | AUCGUG--CCACC | 1.6 |
| 27 | 18.1 | CCGCACGAA | UUCGUG--CCACC | 1.1 |
| 28 | 24-3 | CCGCACGAA | AUCGUG--CCACC | 81.1 |
| 28 | 18.2 | CCGCACUAA | UUAGUG--CCACC | 8.5 |
| 28 | 18.1 | CCGCACGAA | UUCGUG--CCACC | 4.0 |
| 28 | 14.1 | CCGCACAAA | -U-GUG--CCACC | 2.6 |
| 31 | 24-3 | CCGCACGAA | AUCGUG--CCACC | 48.8 |
| 31 | 31.1 | CCGCACACA | AGUGUG--CCACC | 17.8 |
| 31 | 38-6 | CCGCACAAA | AUUGUGCACCACC | 12.0 |
| 31 | 18.1 | CCGCACGAA | UUCGUG--CCACC | 4.8 |
| 31 | 31.2 | CCGCACGGA | ACCGUG--CCACC | 4.2 |
| 31 | 18.2 | CCGCACUAA | UUAGUG--CCACC | 3.6 |
| 31 | 31.3 | CCGCACGAC | GUCGUG--CCAGC | 2.7 |
| 31 | 31.4 | CCGCACGAA | ----------ACC | 1.4 |
| 31 | 18.2 | CCGCACUAA | UUAGUG--CCAAC | 1.2 |
| 31 | 31.5 | CCGCACGAA | ----UG--CCACC | 1.2 |
| 34 | 31.3 | CCGCACGAC | GUCGUG--CCAGC | 46.1 |
| 34 | 38-6 | CCGCACAAA | AUUGUGCACCACC | 44.7 |
| 34 | 31.2 | CCGCACGGA | ACCGUG--CCACC | 3.5 |
| 34 | 31.1 | CCGCACACA | AGUGUG--CCACC | 1.8 |
| 34 | 24-3 | CCGCACGAA | AUCGUG--CCACC | 1.6 |
| 36 | 31.3 | CCGCACGAC | GUCGUG--CCAGC | 53.1 |
| 36 | 38-6 | CCGCACAAA | AUUGUGCACCACC | 42.7 |
| 36 | 31.2 | CCGCACGGA | ACCGUG--CCACC | 1.7 |
| 38 | 38-6 | CCGCACAAA | AUUGUGCACCACC | 50.0 |
| 38 | 31.3 | CCGCACGAC | GUCGUG--CCAGC | 47.0 |
| 38 | 31.2 | CCGCACGGA | ACCGUG--CCACC | 1.3 |
| 43 | 38-6 | CCGCACAAA | AUUGUGCACCACC | 43.5 |
| 43 | 31.1 | CCGCACACA | AGUGUG--CCACC | 38.3 |
| 43 | 31.2 | CCGCACGGA | ACCGUG--CCACC | 9.4 |
| 43 | 31.3 | CCGCACGAC | GUCGUG--CCAGC | 3.2 |
| 46 | 38-6 | CCGCACAAA | AUUGUGCACCACC | 79.4 |
| 46 | 52-2 | CCGCACCAA | AUGGUGCACCACC | 7.4 |
| 46 | 31.1 | CCGCACACA | AGUGUG--CCACC | 3.5 |
| 46 | 46.1 | CCGCACUAA | AUAGUGCACCACC | 2.6 |
| 49 | 52-2 | CCGCACCAA | AUGGUGCACCACC | 75.6 |
| 49 | " | CCGCACCAA | AUGGUGCACCUCC | 17.5 |
| 49 | " | CCGCACCAA | AUGGUGCCCCACC | 3.7 |
| 49 | " | CCGCACCAA | AUGGUGCUCCACC | 1.1 |
| 52 | 52-2 | CCGCACCAA | AUGGUGCACCACC | 80.8 |
| 52 | " | CCGCACCAA | AUGGUGCACCUCC | 9.6 |
| 52 | " | CCGCACCAA | AUGGUGCCCCACC | 3.6 |
| 52 | " | CCGCACCAA | AUGGUGCUCCACC | 1.7 |

Clusters representing >1% of the population in a given round are shown. Clusters that differ by only a single nucleotide outside the P8 stem are given the same name. Clusters that include the wild type, 24-3, 38-6, or 52-2 polymerase are given the name of that polymerase.
