# Supplementary material for: Witnessing the structural evolution of an RNA enzyme
Source: eLife. 2021 Sep 9;10:e71557. doi: 10.7554/eLife.71557 (PMC8460264; doi:10.7554/eLife.71557)
Supplement: Supplementary file 4. — The molecules were synthesized in-house (syn), purchased from IDT (com), or prepared by in vitro transcription (ivt). The T7 RNA polymerase promoter sequence is underlined. Sequences in green indicate the complementary tag on the ribozyme and templates used to improve processivity. Sequences in blue indicate the primer binding site for RNA-templated RNA polymerization. Nucleotides in red are mutations relative to the 52-2 polymerase, that were introduced during PCR assembly. hex, hexynyl group for click chemistry; PEG, polyethylene glycol linker; pcl, photocleavable linker; FAM, 6-fluorescein label; Cy5, cyanine 5-methine label; ppp, 5’-triphosphate added by chemical synthesis. [file elife-71557-supp4.docx]

**Supplementary file 4. Sequences of RNA and DNA molecules used in this study.**

| **Type** | **Name** | **R or DNA** | **Source** | **Sequence (5´→3´)** |
| --- | --- | --- | --- | --- |
| PCR primer | Fwd | DNA | com | GGACTAATACGACTCACTATTAGTCATTGCCGCAC |
|  | Rev | DNA | com | GTCAGCCATGTGTTG |
| Splint | C1 | DNA | com | GACGTATAAGCGTCCGTGCTTTTGCACGTGTGGAGTG |
| Polymerase primer | SP1 | RNA | syn | hex-(PEG)_4_-pcl-PEG-FAM-PEG-GGAGCGAGAA (urea) |
|  | SP2 | RNA | syn | hex-(PEG)_4_-CGCUUAUACGUC-PEG-biotin-PEG-pcl-PEG-FAM-CACUCCACAC (hammerhead) |
|  | P1 | RNA | syn | FAM-biotin-CACUCCACAC (hammerhead) |
|  | P2 | RNA | syn | FAM-biotin-GGAAAAGACAAAUCUGCCCU (ligase) |
|  | P3 | RNA | syn | FAM-biotin-UUGCUACUACACGAC (T1 and R8) |
| Template | urea selection | RNA | ivt | GACAAUGACAAAAAACACUCACACACACUCCACACGGAGAGGUUUCUCGCUC |
|  | hammerhead | RNA | ivt | GACAAUGACAAAAAACGCUUUUCGGCCUUUCGGCCUCAUCAGUACGUCGUGUGGAGUG |
|  | ligase | RNA | ivt | GACAAUGACAAAAAAUCACUAUUGUUGAGAACGUUGGCGUUAAAGCCACCGGGGGCUGCCUCCCCUGCAUCCGAAGAUGUUCUCAAGCUCUGAGGGCAGAUUUGUCUUUUCC |
|  | T1 | RNA | ivt | GACAAUGACAAAAAACACGCUUCGCAGUCGUGUAGUAGCAA |
|  | T5 | RNA | ivt | GACAAUGACAAAAAA‑(CACGCUUCGCA)_5_‑GUCGUGUAGUAGCAA |
|  | T10 | RNA | ivt | GACAAUGACAAAAAA‑(CACGCUUCGCA)_10_‑GUCGUGUAGUAGCAA |
|  | R8 | RNA | ivt | GACAAUGACAAAAAACACUCACACAGGAGAGGUCACUCCACACGUCGUGUAGUAGCAA |
| Ligation | b1-207t | RNA | ivt | GGAAAAGACAAAUCUGCCCUCAGAGCUUGAGAACAUCUUCGGAUGCAGGGGAGGCAGCCCCCGGUGGCUUUAACGCCAACGUUCUCAACAAUAGUGA |
|  | S1 | R/DNA | syn | Cy5-d(AAA)-r(CCAGUC) |
|  | S2 | RNA | syn | pppGGAACAUUAUACGACUGGCACCAU |
| Sequencing | Fwd2 | DNA | com | GGGGGGATGCTACATG |
|  | Fwd3 | DNA | com | TCGTCGGCAGCGTCAGATGTGTATAAGAGACAGCTACAGGGCACTCCACAC |
|  | Fwd4 | DNA | com | TCGTCGGCAGCGTCAGATGTGTATAAGAGACAG GGAAAAGACAAATCTGCC |
|  | Rev2 | DNA | com | ATTGATGGTGCCTACAG |
|  | Rev3 | DNA | com | GTCTCGTGGGCTCGGAGATGTGTATAAGAGACAGATTGATGGTGCCTACAG |
| PCR assembly fragments | F1 (52-2) | DNA | com | GGACTAATACGACTCACTATTAGTCATTGCCGCACCAAGACAAATC |
|  | F1 (G11C) | DNA | com | GGACTAATACGACTCACTATTAGTCATTGCCCCACCAAGACAAATC |
|  | F1 (C12G) | DNA | com | GGACTAATACGACTCACTATTAGTCATTGCCGGACCAAGACAAATC |
|  | F1 (A13U) | DNA | com | GGACTAATACGACTCACTATTAGTCATTGCCGCTCCAAGACAAATC |
|  | F1 (C14G) | DNA | com | GGACTAATACGACTCACTATTAGTCATTGCCGCAGCAAGACAAATC |
|  | F1 (C15G) | DNA | com | GGACTAATACGACTCACTATTAGTCATTGCCGCACGAAGACAAATC |
| PCR assembly fragments | F1 (A16U) | DNA | com | GGACTAATACGACTCACTATTAGTCATTGCCGCACCTAGACAAATC |
|  | F1 (wt) | DNA | com | GGACTAATACGACTCACTATTAGTCATTGCCGCACAAAAAGACAAATCTGCCCTCAGAGC |
|  | F1 (wt/ψknot) | DNA | com | GGACTAATACGACTCACTATTAGTCATTGCCGCACCAAGACAAATCTGCCCTCAGAGCTT |
|  | F2 (52-2) | DNA | com | GTAGATGTTCTCAAGCTCTGAGGGGAGATTTGTCTTGGTGCGGCAA |
|  | F2 (G11C) | DNA | com | GTAGATGTTCTCAAGCTCTGAGGGGAGATTTGTCTTGGTGGGGCAA |
|  | F2 (C12G) | DNA | com | GTAGATGTTCTCAAGCTCTGAGGGGAGATTTGTCTTGGTCCGGCAA |
|  | F2 (A13U) | DNA | com | GTAGATGTTCTCAAGCTCTGAGGGGAGATTTGTCTTGGAGCGGCAA |
|  | F2 (C14G) | DNA | com | GTAGATGTTCTCAAGCTCTGAGGGGAGATTTGTCTTGCTGCGGCAA |
|  | F2 (C15G) | DNA | com | GTAGATGTTCTCAAGCTCTGAGGGGAGATTTGTCTTCGTGCGGCAA |
|  | F2 (A16U) | DNA | com | GTAGATGTTCTCAAGCTCTGAGGGGAGATTTGTCTAGGTGCGGCAA |
|  | F2 (wt) | DNA | com | GCATCCGAAGATGTTCTCAAGCTCTGAGGGCAGATTTGTC |
|  | F3 (52-2) | DNA | com | CAGAGCTTGAGAACATCTACGGATGCAGAGGAGGGGGCCTTCGGTGGATCAA |
|  | F3 (U80G) | DNA | com | CAGAGCTTGAGAACATCTACGGATGCAGAGGAGGGGGCCTTCGGTGGAGCAA |
|  | F3 (C81G) | DNA | com | CAGAGCTTGAGAACATCTACGGATGCAGAGGAGGGGGCCTTCGGTGGATGAA |
|  | F3 (A82U) | DNA | com | CAGAGCTTGAGAACATCTACGGATGCAGAGGAGGGGGCCTTCGGTGGATCTA |
|  | F3 (wt) | DNA | com | TTGAGAACATCTTCGGATGCAGAGGAGGCAGCCTTCGGTGG |
|  | F4 (52-2) | DNA | com | GTGTTGAGAACGGTGGTGCACCATTGATCCACCGAAGGCCCCC |
|  | F4 (C89G) | DNA | com | GTGTTGAGAACGGTGGTCCACCATTGATCCACCGAAGGCCCCC |
|  | F4 (G88C) | DNA | com | GTGTTGAGAACGGTGGTGGACCATTGATCCACCGAAGGCCCCC |
|  | F4 (U87A) | DNA | com | GTGTTGAGAACGGTGGTGCTCCATTGATCCACCGAAGGCCCCC |
|  | F4 (G86C) | DNA | com | GTGTTGAGAACGGTGGTGCAGCATTGATCCACCGAAGGCCCCC |
|  | F4 (G85C) | DNA | com | GTGTTGAGAACGGTGGTGCACGATTGATCCACCGAAGGCCCCC |
|  | F4 (U84A) | DNA | com | GTGTTGAGAACGGTGGTGCACCTTTGATCCACCGAAGGCCCCC |
|  | F4 (U80G) | DNA | com | GTGTTGAGAACGGTGGTGCACCATTGCTCCACCGAAGGCCCC |
|  | F4 (C81G) | DNA | com | GTGTTGAGAACGGTGGTGCACCATTCATCCACCGAAGGCCCC |
|  | F4 (C81G/G88C) | DNA | com | GTGTTGAGAACGGTGGTGGACCATTCATCCACCGAAGGCCCCC |
|  | F4 (A82U) | DNA | com | GTGTTGAGAACGGTGGTGCACCATAGATCCACCGAAGGCCCCC |
|  | F4 (A82U/U87A) | DNA | com | GTGTTGAGAACGGTGGTGCTCCATAGATCCACCGAAGGCCCCC |
|  | F4 (wt) | DNA | com | TTGGGTGTCTGTTGAGAACGTTGG‑‑CGCTATCGCGCCACCGAAGGCTGCCTCCTC |
|  | F4 (wt/ψknot) | DNA | com | TTGGGTGTCTGTTGAGAACGGTGGTGCACCATTGATCCACCGAAGGCTGCCTCCTC |
|  | F5 (52-2) | DNA | com | ACCACCGTTCTCAACACGTACCCGAACATAAAAAGACCTGACAAAAAGGCGATGTTA |
|  | F5 (wt) | DNA | com | CGTTCTCAACAGACACCCAAAAA‑AAAAAGACCTGACGAAAAGGCGATGTTAGA |
|  | F6 (52-2) | DNA | com | GTCAGCCATGTGTTGGGCATGGTACCTGTGCGTGTCTAACATCGCCTTTTTGTCAG |
|  | F6 (wt) | DNA | com | GTCAGCCATGTGTTGGGTATG‑‑ACCTGGGCGTGTCTAACATCGCCTTTTCGTC |

The molecules were synthesized in-house (syn), purchased from IDT (com), or prepared by in vitro transcription (ivt). The T7 RNA polymerase promoter sequence is underlined. Sequences in green indicate the complementary tag on the ribozyme and templates used to improve processivity. Sequences in blue indicate the primer binding site for RNA-templated RNA polymerization. Nucleotides in red are mutations relative to the 52-2 polymerase, that were introduced during PCR assembly. hex, hexynyl group for click chemistry; PEG, polyethylene glycol linker; pcl, photocleavable linker; FAM, 6‑fluorescein label; Cy5, cyanine 5-methine label; ppp, 5´-triphosphate added by chemical synthesis.
