# Supplementary material for: Witnessing the structural evolution of an RNA enzyme
Source: eLife. 2021 Sep 9;10:e71557. doi: 10.7554/eLife.71557 (PMC8460264; doi:10.7554/eLife.71557)
Supplement: Source code 1. [file elife-71557-supp6.zip › Supplementary file 3 - source code 1.docx]

**Supplementary file 2—source code 1.** Python script to identify unique polymerase sequences and number of reads for each from a sequenced round of evolution.

import sys

import string

if len(sys.argv) != 2:

sys.stderr.write("Usage: readcnts.py fastqFile\n")

sys.exit(-1)

# Translation table for reverse complement.

rvtable = string.maketrans("ACGTNacgtn","TGCANtgcan")

reads = {}

readcnt = 0

othercnt = 0

# Read the lines of the FASTQ file.

fp = open(sys.argv[1])

while True:

line1 = fp.readline()

line2 = fp.readline()

line3 = fp.readline()

line4 = fp.readline()

if not line1:

break

if not (line1.startswith("@") and line3.startswith("+") and len(line2) == len(line4)):

sys.stderr.write("Error: Invalid line: %s\n" % line1)

sys.exit(-1)

readcnt += 1

if readcnt % 1000000 == 0:

sys.stderr.write(" -> %d (%d other)\n" % (readcnt, othercnt))

# Extract the read and make its reverse complement.

read = line2.rstrip("\n")[1:-1]

rev = read[::-1].translate(rvtable)

# Currently, just check the first and last four bases to see if the original read

# or its reverse complement should be used in the counting. This could be refactored

# if those bases don't correspond well enough to the actual sequences.

if read.startswith("GACT"):

theRead = read

elif read.startswith("TCAG"):

theRead = rev

elif read.endswith("AGTC"):

theRead = rev

elif read.endswith("CTGA"):

theRead = read

else:

othercnt += 1

continue

# Build a dictionary of the reads, keeping track of the read count for each distinct

# read sequence.

if theRead not in reads:

reads[theRead] = 1

else:

reads[theRead] += 1

fp.close()

# Put the reads and their counts into a list, then sort them by count, and print them.

l = [ (reads[x], x) for x in reads ]

l.sort(reverse=True)

for t in l:

sys.stdout.write(">%d\n%s\n" % (t[0], t[1]))
